# Prospective Evaluation of the Society for Vascular Surgery (SVS) Wound, Ischemia, and Foot Infection (WiFi) Classification for Predicting Amputation Risk in Diabetic Foot Ulcers at a South Indian Tertiary Care Center

**DOI:** 10.7759/cureus.96361

**Published:** 2025-11-08

**Authors:** Seshaan K N B, S Balakrishnan, Karthikeyan Selvaraj

**Affiliations:** 1 Department of General Surgery, Sree Balaji Medical College and Hospital, Chennai, IND

**Keywords:** dfu (diabetic foot ulcer), ischemia, lower limb amputation, risk stratification, society for vascular surgery (svs), tertiary care centers, wifi

## Abstract

Background

Diabetic foot ulcers (DFUs) are a major cause of limb loss. Early identification of high-risk cases is essential, especially in settings where patients often present late and specialized vascular care is limited. The Society for Vascular Surgery (SVS) Wound, Ischemia, and foot Infection (WIfI) classification - which grades severity of the wound, degree of ischemia, and extent of infection - offers a structured approach to assess limb threat and estimate amputation risk. This study aimed to prospectively evaluate the accuracy of the SVS WIfI classification system in predicting lower limb amputation risk among patients with DFUs at a tertiary care center in South India.

Methodology

In this prospective observational study, 50 adult patients presenting with DFUs were evaluated and categorized using the SVS WIfI classification. Patients were stratified into two groups: Group 1 (Stages 1-3) and Group 2 (Stage 4). Demographic, clinical, and laboratory data were collected. Amputation outcomes were assessed, and multivariate logistic regression was performed to identify independent predictors of amputation. Although the estimated sample size was 163, only 50 patients fulfilled eligibility criteria within the study period.

Results

Among the 50 participants, 32 (64%) were classified as WIfI Stage 4. The amputation rate was significantly higher in Group 2 compared with Group 1 (62.1% vs. 6.7%, p < 0.001). Multivariate analysis demonstrated that WIfI Stage 4 was the strongest independent predictor of amputation. Other significant predictors included symptom duration greater than 48 hours, leukocytosis, elevated C-reactive protein (CRP), and poor glycemic control (HbA1c > 8%).

Conclusion

The SVS WIfI classification is a robust and reliable tool for risk stratification in patients with DFUs. Incorporating WIfI staging into routine clinical practice can facilitate timely interventions and help reduce the burden of limb loss, especially in resource-constrained healthcare settings. A relatively small sample size is a limitation; however, the findings reinforce the system’s clinical value and encourage larger-scale validation.

## Introduction

Diabetes mellitus (DM) has emerged as one of the most significant global health challenges, currently affecting more than half a billion adults, with projections exceeding 640 million by 2030 [1]. The long-term burden of DM stems from its chronic complications, which substantially impair daily functioning and reduce survival [1]. Diabetic foot ulcers (DFUs) are among the most severe of these complications, affecting approximately 15-25% of patients with diabetes during their lifetime and accounting for over 80% of diabetes-related lower limb amputations [1,2]. DFUs are associated with high morbidity and mortality, with five-year post-amputation mortality rates reported between 40-70%, comparable to outcomes seen in many advanced malignancies [2]. These alarming statistics highlight the urgent need for early risk stratification and targeted preventive care in patients presenting with DFUs.

The development of DFUs is typically driven by the interplay of peripheral neuropathy, peripheral arterial disease, and infection [2,3]. These risk factors are often compounded by delayed diagnosis, poor glycemic control, and limited access to multidisciplinary care, especially in low- and middle-income countries [3]. Given these challenges, accurate risk stratification tools are essential to identify patients at greatest risk, ensure timely intervention, and improve outcomes in terms of both limb salvage and survival.

Several grading systems have been proposed over the years to assess the severity of DFUs and guide treatment, including Wagner-Meggitt, the University of Texas system, PEDIS, and SINBAD [4]. While these frameworks are useful, they often lack the ability to simultaneously capture the combined effects of wound extent, ischemia, and infection-three crucial determinants of outcome [3,4]. To address this limitation, the Society for Vascular Surgery (SVS) introduced the Wound, Ischemia, and foot Infection (WIfI) classification in 2014 [5]. Modeled after the TNM staging system used in oncology, WIfI assigns independent scores for each component (0-3), which can then be integrated to estimate amputation risk and guide revascularization decisions [5].

The WIfI system has been extensively validated in Western populations, where it has shown strong predictive accuracy for wound healing, risk of amputation, and long-term mortality [6,7]. However, its performance in the Indian setting remains uncertain. Patients in India often present with more advanced disease, have higher rates of uncontrolled diabetes, and face unique challenges such as delayed referral and different microbial profiles of infection. These factors may influence how well the WIfI system predicts outcomes and necessitate local validation before routine use.

This study was therefore designed as a prospective hospital-based investigation in a tertiary care center in South India. Its primary aim was to assess the predictive accuracy of the SVS WIfI classification in determining amputation risk among patients with DFUs. A secondary aim was to identify additional clinical and biochemical predictors - such as symptom duration, inflammatory markers, and glycemic control - that may further improve risk stratification in this population. By achieving these objectives, the study hopes to provide evidence for integrating WIfI into routine clinical practice in India and to support early, targeted interventions that can reduce amputation rates and improve survival among patients with diabetic foot disease.

## Materials and methods

Study design

This hospital-based, prospective observational study was conducted to evaluate the predictive accuracy of the SVS WIfI classification in determining the risk of lower limb amputation among patients with DFUs. The study was carried out in the Department of General Surgery and the Diabetic Foot Clinic at Sree Balaji Medical College & Hospital (SBMCH), Chennai, from January 2024 to December 2024. Each patient was followed for a period of six months after enrollment.

Patient recruitment

Eligible participants were adults aged 15-70 years with a confirmed diagnosis of DM presenting with foot ulcers of diabetic etiology. Exclusion criteria were patients younger than 15 years or older than 70 years, pregnant women, individuals with stump ulcers, and those unwilling to provide informed consent. Eligible participants were recruited using purposive sampling from inpatient wards and outpatient clinics, as DFUs requiring surgical evaluation constitute a distinct high-risk subset. To reduce selection bias, all consecutive eligible patients presenting during the study period were approached for inclusion.

Sample size estimation

Based on preliminary institutional estimates indicating that approximately 64% of DFUs cases present as WIfI Stage 4, the sample size was calculated using the formula for a single proportion with a 95% confidence level and an allowable error of 13%.

Sample size estimation was performeed using the formula for a single population proportion:

n=Zα/22⋅p⋅(1−p)d2n=d2Zα/22​⋅p⋅(1−p)​

where: • n = required sample size • Zα/2​ = 1.96 for 95% confidence level • p = expected proportion of WIfI Stage 4 cases (0.64), based on preliminary institutional data • d = allowable absolute error (0.13)

Substituting these values: n=52.3

Thus, the required sample size was approximately 52 patients. Considering feasibility and anticipated exclusions during the study period, a final target sample size of 50 patients was adopted [6].

Data collection

Baseline demographic, clinical, and laboratory data were collected after written informed consent, including detailed documentation of comorbidities such as hypertension, chronic kidney disease, and coronary artery disease, as well as home medications including antidiabetic, antiplatelet, and vasodilator therapies. Each participant’s ulcer was evaluated using the SVS WIfI classification, wherein wound severity was graded by ulcer size, depth, and tissue loss, ischemia was scored using ankle-brachial pressure index (ABPI) and/or toe pressure according to standardized hemodynamic thresholds (Grade 0: ABPI ≥0.80 or toe pressure ≥60 mmHg; Grade 1: ABPI 0.60-0.79 or 40-59 mmHg; Grade 2: ABPI 0.40-0.59 or 30-39 mmHg; Grade 3: ABPI <0.40 or <30 mmHg), and infection was graded based on clinical evidence of local or systemic involvement. Glycemic control was assessed through fasting and postprandial glucose and glycated hemoglobin (HbA1c). To enable reproducibility of outcome measures, amputations were categorized as minor (distal to the ankle) or major (at or proximal to the ankle). Patients were grouped into WIfI Stages 1-3 versus Stage 4 based on final staging, and all were prospectively monitored for six months via outpatient visits and telephonic follow-up. The six-month period focused on short-term limb-threat outcomes consistent with earlier WIfI validation studies, though limited long-term follow-up is acknowledged as a study limitation.

Primary Outcome

Occurrence of any lower limb amputation (minor or major) within six months.

Secondary Outcomes

Correlation of individual WIfI components with amputation risk and association of glycemic control (HbA1c) with clinical outcomes.

Statistical analysis

Data were entered into Microsoft Excel and analyzed using SPSS version 26 (IBM Corp., Armonk, NY, USA). Categorical variables were expressed as frequencies and percentages and compared using chi-square or Fisher’s exact tests, while continuous variables were presented as mean ± standard deviation (SD) or median (interquartile range), depending on their distribution, and analyzed using independent t-tests or Mann-Whitney U tests. Multivariate logistic regression analysis was performed to identify independent predictors of amputation, with adjusted odds ratios (ORs) and 95% confidence intervals (CIs) reported. A p-value of <0.05 was considered statistically significant.

Ethical considerations

The study protocol was reviewed and approved by the Institutional Human Ethics Committee of SBMCH (Approval No. 002/SBMCH/IHEC/2023/2067). Written informed consent was obtained from all participants prior to enrollment. Patient confidentiality was maintained throughout the study.

## Results

A total of 50 patients with DFUs were enrolled. The cohort was predominantly male, with 36 patients (72.0%), while 14 (28.0%) were female. Most patients were 41-60 years old (n = 29, 58.0%), followed by those >60 years (n = 17, 34.0%), and 15-40 years (n = 4, 8.0%). A diabetes duration of 5-10 years was most common (n = 26, 52.0%), while 12 patients (24.0%) each had diabetes for <5 years and >10 years. Poor glycemic control was prevalent, with 37 patients (74.0%) exhibiting HbA1c > 8%. Based on WHO criteria, 37 patients (74.0%) were overweight or obese. The median BMI was 27.4 kg/m² (IQR: 25.2-30.1). Amputations were required in 21 patients (42.0%), while 29 (58.0%) were managed without amputation. Continuous variables were assessed for normality using the Shapiro-Wilk test and presented as median (IQR), with between-group comparisons conducted using non-parametric tests. Baseline demographic and clinical characteristics are summarized in Table 1.

**Table 1:** Baseline Characteristics of the Study Population (N = 50) Percentages are calculated out of the total study population. HbA1c: glycated hemoglobin; DM: diabetes mellitus; BMI - Body Mass Index

Variable	Frequency (n)	Percentage (%)
Gender		
Male	36	72.0
Female	14	28.0
Age Group		
15–40 years	4	8.0
41–60 years	29	58.0
>60 years	17	34.0
Duration of Diabetes		
<5 years	12	24.0
5–10 years	26	52.0
>10 years	12	24.0
HbA1c		
≤ 8%	13	26.0
> 8%	37	74.0
BMI Category		
<18.5 kg/m²	2	4.0
18.5–24.9 kg/m²	11	22.0
25–29.9 kg/m²	21	42.0
≥30 kg/m²	16	32.0
Amputation		
Yes	21	42.0
No	29	58.0

Analysis of biochemical and clinical severity markers revealed a mean HbA1c level of 9.79% (SD 1.71), with nearly three-quarters of patients showing poor metabolic control. The median wound score was 3, ischemia score 3, and infection score 2, indicating advanced presentation in most cases. The mean WBC count was 18.65 ×10³/mm³ (SD 4.85), reflecting significant systemic inflammation. These clinical and laboratory parameters are summarized in Table 2.

**Table 2:** Distribution of Continuous Clinical and Laboratory Variables (N = 50) Data are presented as mean, standard deviation (SD), median, minimum, and maximum values. HbA1c: glycated hemoglobin; WBC: white blood cell count

Variable	Mean	SD	Median	Min	Max
HbA1c (%)	9.79	1.71	10.2	6.1	12.0
Wound score	2.46	0.88	3.0	1.0	3.0
Ischemia score	2.22	1.17	3.0	0.0	3.0
Infection score	2.04	1.31	2.0	0.0	3.0
WBC (×10³/mm³)	18.65	4.85	20.8	8.1	24.0

When stratified by WIfI staging, 18 patients (36%) were categorized into Group 1 (Stages 1-3: low to moderate limb threat), while 32 patients (64%) were in Group 2 (Stage 4: highest risk). In Group 1, most patients had wound scores of 1-2, ischemia scores of 0-1, and infection scores of 0-2, reflecting less severe disease. Conversely, all Group 2 patients had wound score 3 and infection score 3, consistent with extensive tissue loss and severe infection. This distribution pattern is presented in Table 3.

**Table 3:** Distribution of WIfI Components Between Groups (N = 50) Data represent the distribution of Society for Vascular Surgery WIfI (Wound, Ischemia, and foot Infection) scores across study groups. Group 1: WIfI Stages 1–3; Group 2: WIfI Stage 4.

Component	Group 1 (n = 18)	Group 2 (n = 32)
Wound score		
1–2	13	0
3	5	32
Ischemia score		
0–1	12	0
2	6	0
3	0	32
Infection score		
0–2	14	0
3	4	32

The relationship between WIfI stage and amputation outcomes was striking. Patients in Group 2 (Stage 4) had a significantly higher amputation rate of 62.1%, compared to only 6.7% in Group 1 (Stages 1-3). The relative risk of amputation in Stage 4 was nearly tenfold higher than in earlier stages, with the difference being highly statistically significant (p < 0.001). This strong predictive relationship is shown in Table 4.

**Table 4:** Association of WIfI Stage Groups With Amputation Risk (N = 50) WIfI: Wound, Ischemia, and foot Infection classification. Relative risk is calculated with Stage 1–3 as the reference group.

WIfI Stage Group	Amputation Rate	Relative Risk	p-value
Stage 1–3	6.7%	Reference (1.0)	<0.001
Stage 4	62.1%	~10× higher	<0.001

Multivariate logistic regression identified four independent predictors of amputation. WIfI Stage 4 emerged as the strongest predictor, conferring a tenfold increased risk (OR = 10.0; 95% CI: 4.2-23.8; p < 0.001). Symptom duration greater than 48 hours increased the risk 4.5 times (OR = 4.5; 95% CI: 1.7-11.9; p = 0.002). Elevated WBC (>18,000/mm³) and CRP were also significant, with ORs of 3.2 (95% CI: 1.3-7.9; p = 0.011) and 3.8 (95% CI: 1.5-9.6; p = 0.005), respectively. These findings underscore the impact of delayed presentation and systemic inflammation on limb loss. The predictors of amputation are summarized in Table 5.

**Table 5:** Multivariate Logistic Regression Analysis of Risk Factors for Amputation (N = 50) OR: odds ratio; CI: confidence interval; WIfI: Wound, Ischemia, and foot Infection classification; WBC: white blood cell count; CRP: C-reactive protein

Risk Factor	Adjusted Odds Ratio (OR)	95% CI	p-value
WIfI Stage 4	10.0	4.2–23.8	<0.001
Symptom duration >48 h	4.5	1.7–11.9	0.002
WBC >18,000/mm³	3.2	1.3–7.9	0.011
Elevated CRP	3.8	1.5–9.6	0.005

## Discussion

This prospective study evaluated the predictive accuracy of the SVS WIfI classification in estimating amputation risk among DFU patients in a South Indian tertiary care setting. The findings reinforce WIfI Stage 4 as the strongest determinant of amputation, conferring a tenfold higher risk compared to Stages 1-3 (OR = 10.0; 95% CI: 4.2-23.8; p < 0.001). These results are consistent with international evidence supporting WIfI as a validated tool to stratify limb threat and optimize clinical decision-making [8].

However, the overall amputation rate in our cohort (41.7%) is higher than the 15-25% typically reported from Western centers. This reflects challenges commonly faced in resource-limited environments, including delayed presentation, advanced ischemia, inadequate infection control, and restricted access to specialized multidisciplinary care [9]. Among Stage 4 patients, the amputation rate reached 62.1%, comparable to reports from other low- and middle-income countries [10,11], further highlighting the vulnerability of this group.

Poor metabolic control was evident, with a mean HbA1c of 9.79% and 74% of patients had HbA1c > 8%. Poor glycemic regulation is a recurrent problem in Indian DFU cohorts and is known to impair microvascular function, promote infection, and delay wound healing [12,13]. Coexisting comorbidities such as hypertension, anemia, and chronic kidney disease were also common and may contribute to adverse outcomes. Most patients were on insulin and/or oral hypoglycemic agents, with many also receiving antiplatelets and statins for cardiovascular risk reduction, factors that may modify amputation risk and require further evaluation in future multivariate models [14].

Inflammatory burden further influenced prognosis, with leukocytosis (WBC >18,000/mm³) and elevated CRP emerging as significant predictors of amputation, consistent with earlier studies linking heightened systemic inflammation to extensive tissue necrosis, deep infection, and poor limb salvage [12]. Delayed symptom presentation (>48 hours) increased amputation odds more than fourfold (p = 0.002), reflecting gaps in community awareness and referral pathways-an issue widely reported across India [15].

Clinical integration of WIfI scoring offers a practical solution to improve triage and expedite care. Incorporating WIfI staging into routine workflows at first evaluation-embedded within emergency assessment, surgical clerking, and electronic medical records-can facilitate early identification of high-risk Stage 4 patients who require expedited vascular consultation, aggressive infection management, or urgent admission [15]. Structured referral protocols triggered by predefined WIfI score thresholds could significantly reduce preventable delays in treatment.

This study acknowledges several limitations. The sample size was modest and lower than the initially estimated requirement due to attrition and incomplete ischemia assessment in some potential recruits. The use of purposive sampling may have introduced selection bias, restricting generalizability. Advanced hemodynamic testing, such as TcPO₂ or angiography, was not available for all patients, which may affect ischemia grading accuracy. Additionally, although a six-month follow-up is adequate for short-term outcomes, a longer follow-up is required to capture late ulcer recurrence, re-ulceration, and mortality. These preliminary findings require confirmation through large multicenter studies with comprehensive vascular assessment and extended follow-up durations.

In the context of India’s high DFU burden, the present findings strongly support the routine use of WIfI scoring as an objective, standardized, and resource-appropriate tool for guiding care. Combined with strict glycemic control, targeted infection management, and improved timelines to revascularization when indicated, WIfI-based care pathways can potentially enhance limb salvage in high-risk populations.

## Conclusions

The SVS WIfI classification system is a valuable and practical tool for early risk stratification in DFU patients, demonstrating robust predictive accuracy for amputation risk in this South Indian cohort. WIfI Stage 4 emerged as the strongest determinant of limb loss, underscoring the need for urgent evaluation and aggressive management in these patients. Integration of WIfI-based triage into local hospital protocols, coupled with structured multidisciplinary diabetic foot-care services, may significantly improve clinical outcomes, reduce preventable amputations, and align care delivery in resource-limited settings with established international standards. Further large-scale multicenter studies are warranted to validate these results and strengthen national guidelines for diabetic limb salvage.

## References

[1] Zhang P, Lu J, Jing Y, Tang S, Zhu D, Bi Y (2017). Global epidemiology of diabetic foot ulceration: A systematic review and meta-analysis (†). Ann Med.

[2] Viswanathan V, Madhavan S, Rajasekar S, Chamukuttan S, Ambady R (2005). Amputation prevention initiative in South India: Positive impact of foot care education. Diabetes Care.

[3] Armstrong DG, Boulton AJ, Bus SA (2017). Diabetic foot ulcers and their recurrence. N Engl J Med.

[4] Prompers L, Huijberts M, Apelqvist J (2007). High prevalence of ischaemia, infection and serious comorbidity in patients with diabetic foot disease in Europe. Baseline results from the Eurodiale study. Diabetologia.

[5] Lavery LA, Armstrong DG, Wunderlich RP, Mohler MJ, Wendel CS, Lipsky BA (2006). Risk factors for foot infections in individuals with diabetes. Diabetes Care.

[6] Mills JL Sr, Conte MS, Armstrong DG, Pomposelli FB, Schanzer A, Sidawy AN, Andros G (2014). The Society for Vascular Surgery Lower Extremity Threatened Limb Classification System: risk stratification based on wound, ischemia, and foot infection (WIfI). J Vasc Surg.

[7] Elgzyri T, Larsson J, Thörne J, Eriksson KF, Apelqvist J (2013). Outcome of ischemic foot ulcer in diabetic patients who had no invasive vascular intervention. Eur J Vasc Endovasc Surg.

[8] Jude EB, Oyibo SO, Chalmers N, Boulton AJ (2001). Peripheral arterial disease in diabetic and nondiabetic patients: A comparison of severity and outcome. Diabetes Care.

[9] Lipsky BA, Berendt AR, Cornia PB (2012). 2012 Infectious Diseases Society of America clinical practice guideline for the diagnosis and treatment of diabetic foot infections. Clin Infect Dis.

[10] Abouaesha F, van Schie CH, Griffths GD, Young RJ, Boulton AJ (2001). Plantar tissue thickness is related to peak plantar pressure in the high-risk diabetic foot. Diabetes Care.

[11] Mohan V, Shah S, Saboo B (2013). Current glycemic status and diabetes related complications among type 2 diabetes patients in India: Data from the A1chieve study. J Assoc Physicians India.

[12] Ramachandran A, Snehalatha C, Vijay V, King H (2002). Impact of poverty on the prevalence of diabetes and its complications in urban southern India. Diabet Med.

[13] Joshi SR, Parikh RM (2007). India--diabetes capital of the world: Now heading towards hypertension. J Assoc Physicians India.

[14] Rawshani A, Svensson AM, Rosengren A, Eliasson B, Gudbjörnsdottir S (2015). Impact of socioeconomic status on cardiovascular disease and mortality in 24,947 individuals with type 1 diabetes. Diabetes Care.

[15] Viswanathan V, Rao VN (2013). Problems associated with diabetes care in India. Diabetes Manage.

